# Neural correlates of the inverse base rate effect

**DOI:** 10.1002/hbm.25729

**Published:** 2021-11-26

**Authors:** Angus B. Inkster, Fraser Milton, Charlotte E. R. Edmunds, Abdelmalek Benattayallah, Andy J. Wills

**Affiliations:** ^1^ Brain Research and Imaging Centre University of Plymouth Plymouth; ^2^ University of Exeter Exeter UK; ^3^ Experimental Psychology University of Oxford Oxford UK

**Keywords:** cognitive neuroscience, fMRI, human learning, inverse base rate effect, prediction error

## Abstract

The inverse base rate effect (IBRE) is a nonrational behavioral phenomenon in predictive learning. Canonically, participants learn that the AB stimulus compound leads to one outcome and that AC leads to another outcome, with AB being presented three times as often as AC. When subsequently presented with BC, the outcome associated with AC is preferentially selected, in opposition to the underlying base rates of the outcomes. The current leading explanation is based on error‐driven learning. A key component of this account is prediction error, a concept previously linked to a number of brain areas including the anterior cingulate, the striatum, and the dorsolateral prefrontal cortex. The present work is the first fMRI study to directly examine the IBRE. Activations were noted in brain areas linked to prediction error, including the caudate body, the anterior cingulate, the ventromedial prefrontal cortex, and the right dorsolateral prefrontal cortex. Analyzing the difference in activations for singular key stimuli (B and C), as well as frequency matched controls, supports the predictions made by the error‐driven learning account.

## Graphical abstract

The inverse base rate effect (IBRE) is a nonrational behavioral phenomenon in predictive learning. This study is the first to directly examine the IBRE under an fMRI methodology, noting activations in areas previously linked to prediction error, including the caudate body, the anterior cingulate, the ventromedial prefrontal cortex, and the right dorsolateral prefrontal cortex. Analyzing the difference in these activations for key stimuli supports predictions made by the current best explanation of the IBRE, an error‐driven learning account.

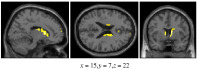

## 1 INTRODUCTION

Learning is a process that enables the use of past and present information to adapt to and overcome present and future challenges. The amount of environmental information present on a moment‐to‐moment basis is large, and so humans have evolved to prioritize the most relevant information. However, the same processes of prioritization can sometimes lead to irrational decisions. The inverse base rate effect (IBRE; Kruschke, 1996, 2001a; Medin & Edelson, 1988; Shanks, 1992) is one example of an irrational decision‐making behavior that seems to occur in this way.

In its canonical form, shown in Table 1, the IBRE involves participants being trained under a simulated medical diagnosis procedure. They are presented with a patient with one of two different pairs of symptoms, and asked to make a judgment, diagnosing the patient with one of two fictitious diseases. For the purposes of this example, we refer to them as “Jominy Fever” and “Phipps Syndrome.” Participants see patients for whom the correct diagnosis is “Jominy Fever” three times as often as those for whom the correct diagnosis is “Phipps Syndrome.” “Jominy Fever” is therefore referred to as the common disease, because its base rate is higher. “Phipps Syndrome” is referred to as the rare disease, due to its lower base rate. The symptom pairs can be considered abstractly as AB and AC. So, a participant might be presented with a patient suffering from “ear aches” and “skin rash” (AB) where the correct diagnosis is “Jominy Fever” (common). They then might see a patient suffering from “ear aches” and “back pain” (AC), with the correct diagnosis being “Phipps Syndrome” (rare). In this example “skin rash” (B) is perfectly predictive of “Jominy Fever” (common), while “back pain” (C) is perfectly predictive of “Phipps Syndrome” (rare). The symptom “ear aches” (A) is uninformative. After being trained in this manner, participants are then presented with both perfectly predictive symptoms, “skin rash” (B) and “back pain” (C). If participants make use of the base rate of the two diseases, they should make the rational diagnosis of the more common disease, “Jominy Fever.” However, the majority of participants preferentially diagnose the patient with the rarer disease, “Phipps Syndrome.” This pattern of responding is called the IBRE.

**TABLE 1:** Canonical IBRE experimental design

Training trials (relative frequency)	Test trials
AB→common (×3)	BC→rare
AC→rare (×1)	

Abbreviation: IBRE, inverse base rate effect.

Currently, the best explanation of the IBRE is the error‐driven learning account implemented within the EXemplar‐based attention to distinctive InpuT (EXIT) formal model (Kruschke, 2001b). Kruschke's error‐driven learning account suggests that, during learning, participants endeavor to reduce the number of errors they make through the shifting of attention. This account predicts that, due to the more frequent occurrence of AB compared to AC, participants learn more about A and B than about C. When they encounter AC, participants initially respond with the common disease due to the presence of A, leading to a prediction error. Attention then shifts away from A and toward C when presented with A and C together, in order to promote new learning and reduce the further occurrence of prediction errors.

EXIT assumes that this attentional reallocation, driven by prediction error, is persistent. As a result, when presented with B and C together during test, the attention to C is greater than the attention to B, resulting in the IBRE. The EXIT model's assumption of the persistence of attentional reallocation is supported by greater eye‐tracking dwell time for C compared to B when presented with BC at test (Kruschke, Kappenman, & Hetrick, 2005). Attention also persists to singly presented cues at test, as demonstrated electrophysiologically by a selection negativity/positivity for C over B when each cue is presented alone on separate test trials (Wills, Lavric, Hemmings, & Surrey, 2014). This attentional persistence to singly presented cues at test is also predicted by EXIT, and is the central prediction investigated in the current study.

One strength of EXIT's error‐driven learning account is that it explains not only the IBRE but also other concurrent response patterns that often occur. When presented with the A cue alone, responding is preferentially common, following the base rate of the two diseases. This is explained by assuming that participants learn to associate A with the common disease more than the rare disease. Another phenomenon occurs when participants are also trained with control cues for B and C, labeled as D and E. These cues are matched for frequency but lack a shared cue (A) during training. This shared‐cue effect is characterized by the IBRE disappearing for the control stimuli, that is, participants do not respond preferentially rare when presented with DE. This has been found in a number of studies (e.g., Kruschke, 2001a; Medin & Edelson, 1988). The error‐driven learning account predicts this effect because, in the absence of a shared cue, there is nothing to cause attentional reallocation on the rare‐outcome trials. While alternative accounts of the IBRE, such as the relative novelty account (Binder & Estes, 1966), and the eliminative inference account (Juslin, Wennerholm, & Winman, 2001) can accommodate the basic IBRE, they fail to account for the shared‐cue effect (Kruschke, 2001a; Wills et al., 2014).

The only previous published fMRI study of the IBRE was conducted by O'Bryan, Worthy, Livesey, and Davis (2018). They made use of an atypical IBRE procedure involving real‐world visual categories (scenes, faces and objects) as stimulus features to allow their use of multivoxel pattern analysis (MVPA). While this approach was well motivated, one consequence of this atypical procedure was the lack of a compelling behavioral IBRE in their study. Specifically, the defining feature of the IBRE is the presence of greater rare than common responses to BC. O'Bryan et al. report the presence of a numerical effect in that direction, without reporting inferential statistics for this contrast; our analysis of their raw data indicates Bayesian evidence for the absence of the IBRE in their study, ${\mathrm{BF}}_{10}=.27$. The inferential tests reported by O'Bryan et al. provide evidence for base‐rate neglect rather than the IBRE.^1^

In the current study, we employed a more standard procedure from our previous work, known to robustly demonstrate the IBRE (Inkster, 2019; Wills et al., 2014). We had two predictions, based on EXIT, the leading account of the IBRE, and on our previous electrophysiological work (Wills et al., 2014). Our first prediction, well supported in general terms by previous neuroimaging work on the correlates of prediction error, was that the striatum (comprising the caudate nucleus, the putamen, and the nucleus accumbens), the medial anterior prefrontal cortex, and the anterior cingulate would show more activation for AC than for AB during training. This is because AC results in more prediction errors than AB behaviorally, and because previous work, including two major meta‐analyses (Fouragnan, Retzler, & Philiastides, 2018; Garrison, Erdeniz, & Done, 2013), implicate these areas in the processing of prediction errors. There is also good evidence that the right dorsolateral prefrontal cortex is involved in the processing of prediction errors (Fletcher et al., 2001; Fouragnan et al., 2018; Turner et al., 2004). We thus defined a region of interest (ROI) for all of our analyses that comprised these four areas.

As discussed by Fouragnan et al. (2018), the activity in brain areas associated with prediction error is likely due to a number of different processes, including outcome valence processing, attentional processing—sometimes described as “surprise” processing or the modulation of associability (Mackintosh, 1975; Pearce & Hall, 1980)—as well as the calculation of signed prediction error that is most commonly associated with the term *prediction error* (and as instantiated by, e.g., the Rescorla–Wagner (Rescorla & Wagner, 1972) and temporal difference models (Sutton & Barto, 1987).

Our second prediction for the current study concerns the possible attentional‐processing role of prediction‐error‐associated brain areas, and comes from the EXIT model's explanation of the IBRE. A key part of EXIT's architecture is a back‐propagation process, driven by prediction error, which adjusts future attention to stimuli in order to minimize errors. In the case of the IBRE procedure, when participants encounter AB, attentional changes are less frequent due to both A and B being associated with the common outcome and so there is less chance of an error being made (B because of it being a perfect predictor of the common outcome, A because it occurs more frequently with the common outcome than the rare). When they encounter AC, errors are dependent on the cue preferentially attended to and are more frequent, due to the disjoint of C being a perfect predictor of the rare outcome and A being associated more heavily with the common outcome. On these trials, EXIT predicts that when a prediction error occurs, cue attention for future AC trials is shifted such that more attention is paid to the C cue. The model assumes that these attentional changes are persistent, and that in order for the IBRE to occur this attentional reallocation persists into the test phase, producing the preferential rare outcome responding to BC at test (i.e., because C is attended more than B). Previous eye‐tracking and neuroscience work (Kruschke et al., 2005; Wills et al., 2014; Wills, Lavric, Croft, & Hodgson, 2007) observed these persistent attentional changes, and other work (Fouragnan et al., 2018) acknowledge the possibility that other neuroscience studies of prediction error could be observing persistent attentional changes caused by prediction error; rather than (or as well as) the initial computation of prediction error.

In the context of the current study, our prediction is that this persistence of attentional reallocation would manifest as greater activation for cue C, presented alone at test, than for cue B, presented alone at test. Our assumption that attentional reallocation persists not only into the test phase, but also to singly presented cues is supported by our previous neurophysiological work (Wills et al., 2014). Thus, our a priori prediction was that we would see greater activation for C than for B during test in our prediction‐error ROI. If confirmed, this prediction would further support the EXIT account of the IBRE, and would suggest that the brain areas in which this difference was observed may be involved in the persistent attentional reallocation that can occur in response to prediction errors.

## 2 METHODS

### 2.1 Participants

Thirty‐four people were recruited from the University of Exeter participant pool. Participants received either course credit or £10. Participants gave informed consent according to procedures approved by the Psychology Ethics Committee, University of Exeter. Five participants' data were removed due to excessive head movements during the experiment, rendering their fMRI data unusable. Participants' accuracy in the final block of training was then assessed using a learning criterion. This criterion was identical to the one used in Wills et al. (2014), where participants scoring less than 72% in the final block of training were excluded from further analysis. This criterion represents the level of accuracy that cannot be attributed to random responding based on the block length of 18 trials. Applying this criterion necessitated the removal of four participants, resulting in a final data set of 25 participants. Participants for this study were recruited with no specific exclusion on the basis of age, sex, or race.

### 2.2 Procedure

**FIGURE 1:**
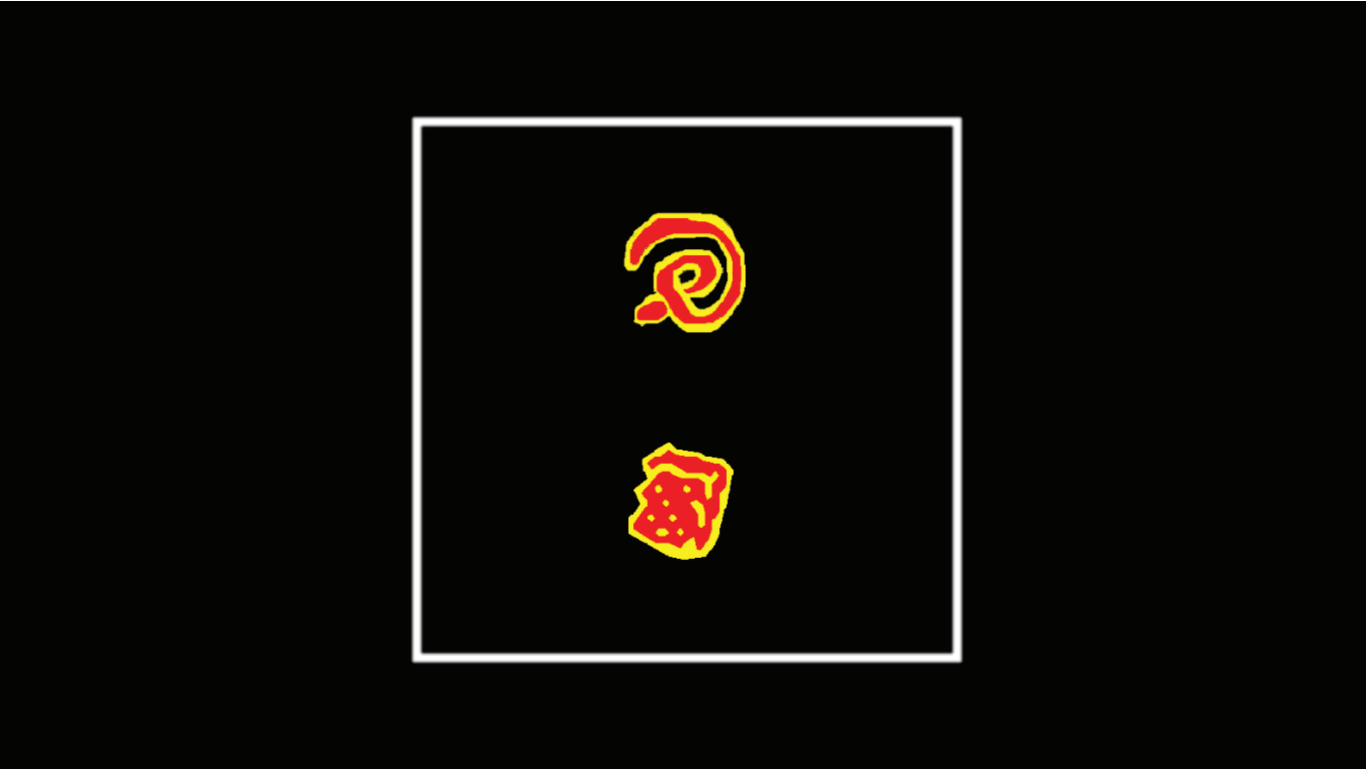
An example trial

The abstract design and stimuli are identical to that of Wills et al.'s (2014) electrophysiological study, and can be seen in Table 2 and Figure 1 respectively. The stimuli are abstract shapes, referred to as “cells” due to the context of the experiment; a medical diagnosis task. The ratio of common to rare in this design (2:1) differs from the ratio in the canonical IBRE design (3:1). The reason for this is the same as in Wills et al.; it shortens study duration in order to avoid participant fatigue, given the necessarily long test phase required for a neuroscience study. Previous work (Inkster, 2019; Wills et al., 2014) has shown that a robust IBRE can be achieved with a 2:1 ratio of common to rare.

**TABLE 2:** Experimental design

Training trials (relative frequency)	Test trials	
$A_{1}B_{1}\to \text{common}$ (×2)	A_1_B_1_, A_2_B_2_, A_3_B_3_	×4
$A_{2}B_{2}\to \text{common}$ (×2)	F_1_D_1_, F_2_D_2_, F_3_D_3_	
$A_{3}B_{3}\to \text{common}$ (×2)	A_1_C_1_, A_2_C_2_, A_3_C_3_	×2
$A_{1}C_{1}\to \text{rare}$ (×1)	G_1_E_1_, G_2_E_2_, G_3_E_3_	
$A_{2}C_{2}\to \text{rare}$ (×1)	B_1_, B_2_, B_3_	×5
$A_{3}C_{3}\to \text{rare}$ (×1)	C_1_, C_2_, C_3_	
$F_{1}D_{1}\to \text{common}$ (×2)	D_1_, D_2_, D_3_	
$F_{2}D_{2}\to \text{common}$ (×2)	E_1_, E_2_, E_3_	
$F_{3}D_{3}\to \text{common}$ (×2)	A_1_, A_2_, A_3_	
$G_{1}E_{1}\to \text{rare}$ (×1)	B_1_C_1_, C_2_C_2_	
$G_{2}E_{2}\to \text{rare}$ (×1)	B_3_C_3_, D_1_E_1_	
$G_{3}E_{3}\to \text{rare}$ (×1)	D_2_E_2_, D_3_E_3_	

*Note*: Each abstract stimulus is represented by three “cells” randomized between participants. The subscripted numbers represent the specific “cell” tied to the abstract stimulus present on a trial. Example “cells” can be seen in Figure 1.

In each phase of the experiment, trial order was randomized. Participants were asked to take on the role of a doctor, diagnosing patients with either “Jominy Fever” or “Phipps Syndrome” on the basis of the “cells” they were presented with. These instructions were given prior to them entering the scanner. The response key that represented each disease was also explained to the participant before the task began, and was counterbalanced between participants. The disease that was abstractly common or rare was also counterbalanced. The mapping between cues and outcomes was deterministic, for example, A_1_B_1_ was always followed by the common disease, and A_1_C_1_ was always followed by the rare disease.

The experiment was displayed on a back‐projection screen positioned at the foot end of the MRI scanner and viewed via a mirror mounted on a head coil. Button‐press responses and reaction times (RTs) were measured using a fiber‐optic button box. The training phase consisted of 10 blocks of 36 trials, making 360 trials in total. Each trial began with a variable duration fixation cross presented in the center of the screen. The durations were generated using an exponential distribution, following the method described in Haberg, Zito, Patria, and Sanes (2001). The range of the durations was 250–3,500 ms, with a mean duration of 1,284 ms.

After the fixation cross, a gray view box was displayed on its own for 500 ms to indicate where the stimuli would appear. The “cell” stimuli appeared toward the top and bottom of the view box, with location randomized on each trial. The cells remained on screen for 2,000 ms, during which time participants made their diagnosis using either the left or right button on the button box. After this, participants received corrective feedback for 500 ms which included naming the correct diagnosis. If a response was not made within 2,000 ms, participants instead received a time‐out message.

Further instructions were given at the start of the test phase. Participants were informed that they would still diagnose patients and would see some cells that they had seen before, continuing to receive feedback for these cells. These were the same cue compounds presented during training, and were presented in the same ratio as in training. The first four rows of the test trials column in Table 2 represent these trials. Training trials for which participants received corrective feedback in the test phase are not always included in IBRE procedures, but this approach addresses the potential concern that performance will deteriorate over the course of the necessarily lengthy test phase, by providing additional learning in order to stabilize performance. This technique was employed successfully in both Wills et al. (2007) and Wills et al. (2014).

Participants were further told that they would see some cell combinations that they would not receive feedback for. These trials were novel to the test phase, and can be seen in the test trials column in Table 2 (row five onward). The test phase consisted of 282 trials in total. The number of test trials was constrained such that the key test stimuli (B, C, D, E) were presented enough to adequately power the fMRI analyses, but that the test phase was not excessively long, so as to avoid participant fatigue.

The trial structure in the test phase was the same as in the training phase, but with the addition of single cells being presented in the center of the view box. The variable duration of the fixation cross had the same range of times as in the training phase, and a similar mean duration of 1,226 ms.^2^ On trials for which participants did not receive feedback, they instead received the message “DATA MISSING” and a series of question marks.

### 2.3 Analysis of behavioral data

Trials where participants timed out were removed from further analysis and constituted less than 1% of the total number of trials across all participants. In addition to conventional null‐hypothesis tests, we also calculated Bayes factors (BF) for theoretically central analyses. These were calculated using the procedure recommended by Dienes (2011), implemented within an R script by Baguley and Kaye (2010). Predicted differences were estimated from a behavioral‐only version of the same experiment previously run in our lab (Experiment 3; Inkster, 2019). As recommended by Dienes, we assumed a half‐normal distribution for the prior with a mean of zero and a *SD* equal to the predicted difference. By convention, where BF > 3, the experiment has found evidence for the alternative hypothesis, whereas if BF < 1/3, the experiment finds evidence for the null hypothesis (Jeffreys, 1961). Values between a third and three are generally considered inconclusive, although they still carry information. For example, where BF = 2, this tells us that the experimental hypothesis is now about twice as likely as it was before we conducted the experiment.

### 2.4 fMRI data acquisition

Images were collected using a 1.5‐T Gyroscan magnet equipped with a Sense coil (Philips, Amsterdam, The Netherlands). A T2*‐weighted echo‐planar sequence was used (repetition time = 3,000 ms, echo time = 45 ms, flip angle = 90°, 32 transverse slices, field of view = 240 mm, 3.5 × 2.5 × 2.5 mm). The training phase comprised two runs of 242 scans, and the test phase two runs of 187 scans. Standard volumetric anatomical MRI was performed after functional scanning by using a 3D T1‐weighted pulse sequence (repetition time = 25 ms, echo time = 4.1 ms, flip angle = 30°, 160 axial slices, 1.6 × 0.9 × 0.9 mm).

### 2.5 Analysis of fMRI data

Analyses were carried out using SPM12 software (FIL Methods Group, 2014). Functional images were corrected for acquisition order, realigned to the mean image, and resliced to correct for motion artifacts. The realigned images were coregistered with the structural T1 volume, and the structural volumes were spatially normalized. The spatial transformation was applied to the realigned T2* volumes, which were spatially smoothed using a Gaussian kernel of 8 mm FWHM. Data were high‐pass filtered (1/128 Hz) to account for low‐frequency drifts. The BOLD response was modeled by a canonical hemodynamic response function with temporal and dispersion derivatives.

In the individual participant models, the critical trials for comparisons (AB and AC for the training phase; B, C, D, E, BC, and DE for the test phase) were included as individual regressors, with the other, noncritical, trial types and time‐outs included as two further separate regressors of no interest. The duration of each event was modeled as the participant's RT for that trial, an approach advocated in Grinbrand, Erdeniz, Lindquist, Ferrera, and Hirsch (2008).

Our three principal analyses were conducted on comparisons of singly presented cues in the test phase; these principal analyses were: comparing C‐B, comparing E‐D and the critical analysis, comparing the levels of activation in the previous two comparisons; (C‐B)–(E‐D). The C‐B comparison is a direct examination of our central prediction that activations in brain regions linked to prediction error would be greater for C presented alone, relative to B presented alone. The E‐D comparison is similar to the C‐B comparison but has a different purpose. E and D serve as frequency matched controls to C and B, so any difference in the comparisons must be due to the presence or absence of the shared cue during training. The (C‐B)–(E‐D) comparison provides a direct test of these differences.

In addition to our principal analyses, we also conducted two further analyses. The first of these compared activation linked to AC and AB in brain areas previously linked to prediction error (and thus included in our ROI) in our training phase fMRI data. From both the behavioral data, and from the EXIT model, it is possible to predict that there will be more prediction errors on AC trials than AB trials, and hence areas associated with prediction error should be more active on AC trials than AB trials. The second of our additional analyses compared activation linked to BC and DE in our ROI during the test phase. EXIT does not predict a difference between these two compound cues; it instead predicts that the way attention is distributed between the cues within the compounds is the key difference. Nonetheless, as BC is the key behavioral cue, an obvious comparison to make is between BC and its frequency‐matched control compound, DE. A further justification for this contrast is that theories other than EXIT might predict a neural difference between these two compounds.

The mask used for the ROI analysis was constructed using the WFU Pickatlas (Maldjian, Laurienti, Burdette, & Kraft, 2003), and was comprised of the brain regions we predicted to be linked to prediction error in our Introduction. Specifically, these regions were the striatum (bilateral caudate, putamen and nucleus accumbens), the right dorsolateral prefrontal cortex (BA 9 and BA 46), the medial anterior prefrontal cortex (BA 9 and BA 10) and the anterior cingulate (BA 24, BA 32, and BA 33). The number of voxels within this mask was 11,952. Alongside ROI analysis, we also conducted exploratory whole brain analysis for each of the above comparisons.

The fMRI analyses were completed using a hierarchical general linear model, with first‐level analyses conducted at the individual subject level and second‐level analyses at the group level using a random effects model. The ROI analyses were conducted with a combined statistical threshold of p<.005 and the following thresholds of contiguous voxels: 30 for the training phase analyses and 26 for the test phase analyses. These thresholds together produce an overall corrected threshold of p<.05; based on cluster‐level inference corrected for familywise error rate according to cluster size. These values were estimated using AlphaSim as implemented in the REST toolbox (Version 1.8, Song et al., 2011). For these calculations, smoothness was estimated within SPM12 using the group residuals from the general linear model and were 9.0 × 9.0 × 8.8 mm for the training phase and 9.7 × 9.7 × 9.4 mm for the test phase.

The test phase whole brain analyses were conducted with a combined statistical threshold of p<.001 and 110 contiguous voxels. These thresholds together produce an overall corrected threshold of p<.05; again based on cluster‐level inference corrected for familywise error rate according to cluster size. These values were again estimated using Alphasim (REST, Version 1.8, Song et al., 2011). For all analyses, normalized MNI space coordinates were transformed to Talairach space using GingerALE (Eickhoff et al., 2011) and assigned anatomical labels using the Talairach Client (http://talairach.org/client.html) as per the atlas of Talairach and Tournoux (1988).

## 3 RESULTS

### 3.1 Behavioral analyses

**FIGURE 2:**
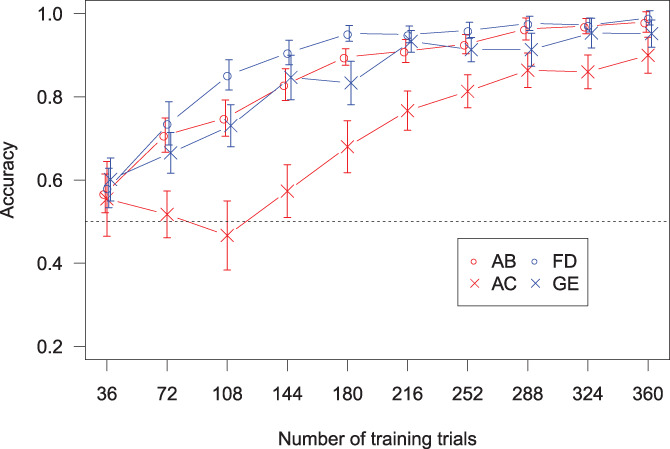
Training accuracy. The error bars are within‐subject Cousineau–Morey 95% confidence intervals

The accuracy of participants across the training phase is shown in Figure 2. A three‐way analysis of variance (ANOVA) was conducted on the training phase data, looking at the effects of training block (first/last), stimulus frequency (common/rare), and shared cue (present/not present) on accuracy. Accuracy in the final block was significantly higher than the first block, $F\left(1,24\right)=324.63,p<.001$. No other significant main effects or interactions were found.

A further two‐way ANOVA was conducted on the data in the final block of training, looking at the effects of stimulus frequency and shared cue on accuracy. Accuracy was significantly higher for the common stimulus compounds (AB and FD) than for the rare stimulus compounds (AC and GE), $F\left(1,24\right)=5.23$, p=.03. No other significant main effects or interactions were found.

Table 3 shows the response proportions for each of the stimuli presented in the test phase. The IBRE test stimulus BC was found to have a significantly greater proportion of rare responses than .5, ${\mathrm{BF}}_{10}=31,t\left(24\right)=2.93,p=.003$. Given there are only two response options in the current experiment, this demonstrates the presence of an IBRE. The proportion of common responses to the A stimulus was significantly greater than .5, as expected, $t\left(24\right)=6.14,p<.001$. Also as expected, there were fewer rare responses to DE than to BC, although the evidence for this difference was inconclusive, ${\mathrm{BF}}_{10}=1.8,t\left(24\right)=1.57,p=.07$.

**TABLE 3:** Proportion of responses to each of the stimulus types presented in the test phase

Stimulus type	Common	Rare
A	.76 (*.76*)	.24 (*.24*)
AB	.92 (*.93*)	.08 (*.07*)
AC	.19 (*.17*)	.81 (*.83*)
B	.92 (*.90*)	.08 (*.10*)
**BC**	.35 (*.36*)	**.65** (*.64*)
C	.15 (*.15*)	.85 (*.85*)
D	.85 (*.86*)	.15 (*.14*)
**DE**	.44 (*.43*)	**.56** (*.57*)
E	.24 (*.24*)	.76 (*.76*)
FD	.96 (*.94*)	.04 (*.06*)
GE	.11 (*.13*)	.89 (*.87*)

*Note*: Bold indicates the behavioral results analyzed. Values within brackets (italics) are simulated response proportions from the EXIT model.

Abbreviation: EXIT, EXemplar‐based attention to distinctive InpuT.

Table 3 further shows the response proportions produced by the EXIT formal model (Kruschke, 2001b), within brackets next to the behavioral data. As can be seen from the table, EXIT provides an extremely close fit to the behavioral data, capturing the response patterns for each stimulus, $\text{RMSD}=.01,r^{2}>.99$. For technical details of our simulation methodology, see Appendix.

### 3.2 Imaging analyses

#### 3.2.1 Training phase

**FIGURE 3:**
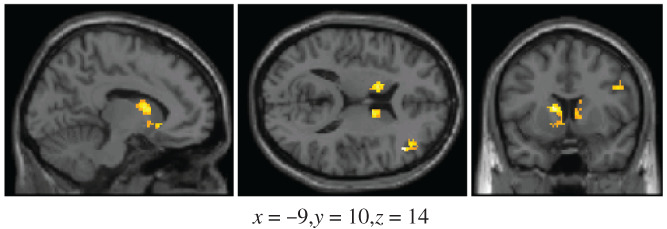
Areas that show greater activation for the AC cue compound compared to the AB cue compound under a region of interest (ROI) analysis, during the training phase. The thresholds used were p<.005 and 30 contiguous voxels

We first compared AC with AB in our ROI, during the training phase. This analysis revealed a number of brain regions that exhibited greater activations for AC compared to AB (see Figure 3). These regions were the bilateral caudate body (peak cluster size: 214, peak voxel *x* = −14, *y* = 7, *z* = 15) and the right dorsolateral prefrontal cortex (BA 9; peak cluster size: 41, peak voxel *x* = 43, *y* = 5, *z* = 32).

#### 3.2.2 Test phase

##### BC‐DE comparison

The EXIT model does not predict a difference between these two compound cues, because it is the distribution of attention within the compound that is predicted to vary between the two compounds, not the total amount of attention to BC versus DE. Specifically, C is predicted to be more attended than B, while attention should be more evenly distributed between D and E. As expected, no significant differences were found, either in ROI or whole‐brain analyses.

##### C‐B comparison

**FIGURE 4:**
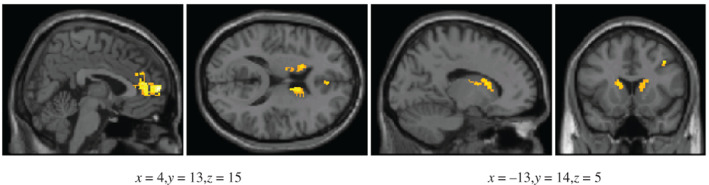
Areas that show greater activation for the C stimulus compared to the B stimulus during the test phase, under a region of interest (ROI) analysis. The thresholds used were p<.005 and 26 contiguous voxels

The ROI analysis revealed a number of brain regions that exhibited greater activations for C (stimulus associated with the rare outcome) than for B (stimulus associated with the common outcome), see Figure 4 and Table 4. These regions included the ventromedial prefrontal cortex (BA 10), medial prefrontal cortex (BA 9), right dorsolateral prefrontal cortex (BA 9), bilateral caudate body, and left anterior cingulate (BA 32).

**TABLE 4:** Brain regions activated during the test phase for an ROI analysis of C‐B. The thresholds used were p<.005 and 26 contiguous voxels

Region	Cluster size		Talairach coordinates			*z*‐Score
		BA	x	y	z	
Right ventromedial prefrontal cortex	219	10	3	55	17	4.46
Right anterior cingulate		32	3	39	15	3.88
Right medial prefrontal cortex		9	3	48	18	3.58
Right caudate body	226		8	1	14	3.97
Right caudate body			12	−17	21	3.51
Right caudate body			14	2	23	3.12
Right dorsolateral prefrontal cortex	32	6	34	6	41	3.43
Right dorsolateral prefrontal cortex		9	39	10	38	2.82
Left caudate body	135		−8	1	10	3.26
Left caudate body			−16	8	17	3.24
Left caudate body			−12	14	13	3.08
Left anterior cingulate	58	32	−8	41	10	3.09
Left ventromedial prefrontal cortex		10	−3	52	13	2.69

Abbreviation: ROI, region of interest.

A number of brain areas included in the ROI analysis were also activated under whole brain analysis including a cluster comprising the right medial frontal cortex and the anterior cingulate (cluster size: 228, peak voxel *x* = 3, *y* = 55, *z* = 17). Outside of brain areas already identified in the ROI analysis the right thalamus was activated (cluster size = 257, peak voxel x=12,y=−11,z=18), as well as a separate cluster in the left cerebellum (cluster size = 111, peak voxel x=−25,y=−70,z=−28).

##### E‐D comparison

The E‐D comparison differs from the previous comparison in one key respect; the absence of a shared cue presented alongside E and D in training. Given the predictions of the error‐driven learning account, and previous work (Kruschke, 2001a; Wills et al., 2014), we would expect to see no difference in activations here.

An ROI analysis examined activations for the E stimulus compared to the D stimulus and failed to find any areas that showed a significant difference in activation. Although this is unsurprising theoretically, these analyses were conducted to both stay consistent with the previous comparison and to characterize this comparison given its use in the final, critical, comparison. Whole brain analysis also failed to show any areas with a significant difference in activation.

##### (C‐B)–(E‐D) comparison

This comparison is the critical analysis for the current experiment. The previous test phase comparisons differ in one key way; the presence or absence of a shared cue when training with those stimuli. While any difference in the areas of the brain activated between these comparisons can be attributed to this factor, the (C‐B)–(E‐D) comparison provides a direct test of this difference; and so rule out any novelty‐based explanation of the activations noted in the C‐B comparison.

**FIGURE 5:**
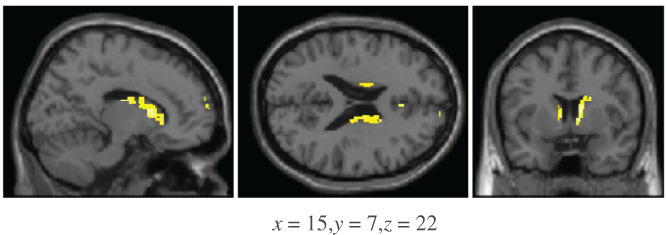
Areas that show greater activation for the C‐B comparison compared to the E‐D comparison under a region of interest (ROI) analysis, during the test phase. The thresholds used were p<.005 and 26 contiguous voxels

An ROI analysis revealed a number of brain regions exhibiting greater activation for the C‐B comparison compared to the E‐D comparison (Figure 5 and Table 5). Greater activation was noted in the bilateral caudate, the bilateral anterior cingulate, the right superior prefrontal cortex, and right ventromedial prefrontal cortex.

**TABLE 5:** Brain regions activated during the test phase for the ROI analysis of the comparison of the C‐B comparison and the E‐D comparison. The thresholds used were p<.005 and 26 contiguous voxels

			Talairach coordinates			
Region	Cluster size	BA	x	y	z	*z*‐Score
Right caudate body	395		8	1	14	3.86
Right caudate body			10	9	11	3.75
Right caudate body			6	6	5	3.55
Left caudate body	36		−8	1	10	3.45
Right anterior cingulate	32	24	4	21	24	3.39
Right anterior cingulate		24	4	29	18	3.06
Right superior prefrontal cortex	45	9	8	56	24	3.35
Right ventromedial prefrontal cortex		10	5	56	13	2.94
Left anterior cingulate	47	32	−8	39	11	3.17
Left anterior cingulate		32	−6	45	7	2.98
Left caudate body	32		−14	−11	19	3.13
Left caudate body	48		−16	8	17	3.05
Left caudate body			−16	16	15	2.75

Abbreviation: ROI, region of interest.

The whole brain analysis also identified two clusters outside the areas identified in the ROI analysis, in the right thalamus (cluster size = 125, peak voxel x=4,y=−19,z=12) and the left cerebellum (cluster size = 155, peak voxel x=−29,y=−68,z=−30).

## 4 DISCUSSION

The IBRE is a nonrational phenomenon in which people, having learned that cue compound AB predicts a common disease and cue compound AC predicts a rare disease, go on to predict that BC predicts the rare disease, in opposition to the underlying base rates (Kruschke, 1996; Medin & Edelson, 1988; Shanks, 1992). The current study was the first investigation of a successfully‐observed IBRE with fMRI.

We made a number of predictions about brain activity and investigated them using ROI analysis. The predictions were made on the basis of: (one) an error‐driven learning account of the IBRE, expressed as a formal model (Kruschke, 2001b), (two) a previous electrophysiological study of the IBRE (Wills et al., 2014), and (three) a substantial body of previous work on the neural correlates of prediction error (e.g., Fouragnan et al., 2018).

As predicted, a number of brain regions previously associated with prediction error during training showed greater activation during the test phase for the C cue relative to the B cue. These regions included the ventromedial prefrontal cortex, medial prefrontal cortex, right dorsolateral prefrontal cortex, bilateral caudate body and left anterior cingulate. A number of previous studies have linked these areas to the occurrence of prediction error (e.g., Fletcher et al., 2001; Fouragnan et al., 2018; Garrison et al., 2013; Turner et al., 2004). These differences were not detectable for the frequency‐matched control cues D and E, which were presented in training without the shared cue A. Greater activations were also noted in the right dorsolateral prefrontal cortex and bilateral caudate body during the training phase for the AC cue relative to the AB cue; a result consistent with both previous work and our test phase analysis.

Taken together, these results provide strong evidence in support of the prediction‐error‐based account of the IBRE (Kruschke, 2001b). Specifically, the current results, alongside those of Kruschke et al. (2005) and Wills et al. (2014), support the idea that the effects of prediction error during training persist into the test phase, and can be observed in singly presented cues. These differences are characterized in EXIT as persistent changes in attentional allocation, and this characterization in turn supports the idea that activity in brain areas associated with prediction error is sometimes associated with differences in attentional processing. Further support for Kruschke's account of the IBRE comes from the excellent level of quantitative fit of his EXIT model to the behavioral data of the present study (see Table 3).

Exploratory whole‐brain analysis of the test phase identified several additional brain areas that might be involved in the IBRE. These areas were not predicted in advance so any inferences must be treated with some caution. One area in the thalamus showed a difference in activation for the C cue relative to the B cue. Given its role in relaying and processing sensory information (Schiff, 2008), its activation in this task is not unexpected. Another area in the left cerebellum also showed a difference in activation for the C cue relative to the B cue. This is perhaps unsurprising given that this area has been implicated in a wide range of cognitive tasks including learning (Desmond & Fiez, 1998); such as a previous category learning experiment (Carpenter, Wills, Benattayallah, & Milton, 2016).

There was some overlap between the areas of activation observed in the present work, and those observed in the only previous attempt to study the IBRE with fMRI (O'Bryan et al., 2018). O'Bryan et al. reported activations in the PFC, thalamus and cerebellum; areas also identified in our key contrast. Direct comparison of the two studies is difficult, however, due to differences in analysis methodology. The analyses conducted in the current study are direct stimulus contrasts, while O'Bryan et al. correlated brain activity with internal values of the dissimilarity‐based extension of the generalized context model (Stewart & Morin, 2007). Nevertheless, the overlap in some of the regions identified across the studies is intriguing, even with this caveat in mind.

Inferring from this overlap should be approached with some caution though, as O'Bryan et al.'s (2018) conclusions appear somewhat different to those of the current study, and to those of a number of previous experiments on the IBRE. A key conclusion from O'Bryan et al.'s MVPA is that, on trials where participants respond rare to BC, they process B *more* intensively than C. O'Bryan et al. note that eye‐tracking would be a good way to corroborate this finding; a methodology previously employed in the study of a variant of the IBRE by Kruschke et al. (2005). Kruschke et al. reported *less* attention to B than C on BC trials when an IBRE was observed, a finding further supported by the ERP results of Wills et al. (2014). Nonetheless, future work on the IBRE should further consider the theoretical implications of both sets of results.

In the current work, we have focused on the predictions of the EXIT model, as these were the a priori basis of our experiment. Other formal models of category learning are available. One particularly pertinent alternative in the current case, given its predictions about the relationship between cognitive and neural processes, is the COmpetition between Verbal and Implicit Systems (COVIS) model (Ashby, Alfonso‐Reese, Turken, & Waldron, 1998). We note that one of the areas identified in our key contrast was the caudate body, to which COVIS attributes stimulus representation in the procedural learning system. Nomura et al. (2007) suggest that feedback‐driven learning strengthens synapses in the caudate through a reward signal, and the idea that the caudate is involved in some kind of associative learning process is consistent with a number of other related results (e.g., Carpenter et al., 2016; Seger & Cincotta, 2005). The COVIS procedural system, in its current form, does not provide an explanation for the IBRE, but it could potentially be modified to do so by the inclusion of the sort of error‐driven attentional‐allocation process employed in EXIT and investigated in the current work.

While we argue for the role of prediction error in the brain regions identified in our analysis, it is worth acknowledging that some of these areas, in particular the DLPFC, have been linked to other cognitive processes. Schlösser et al. (2009) evidenced a link between DLPFC and the processing of uncertainty; clearly, this could play a role in the handling of the BC test cue, due to uncertainty generated as a result of the conflicting information provided by the B and C cues individually. Similarly, Badre and D'Esposito (2007, 2009) link the lateral PFC to hierarchical cognitive control processes, including attentional control. This is interesting, as EXIT arguably instantiates a controlled process of attentional reallocation; for example, it has previously been proposed that concurrent load disables attentional reallocation in this kind of model (Nosofsky & Kruschke, 2002).

### 4.1 Conclusion

The current study provides the first evidence linking the bilateral caudate body, left anterior cingulate, right dorsolateral prefrontal cortex, ventromedial prefrontal cortex, and medial prefrontal cortex to the IBRE. These neural correlates are strongly linked to the occurrence of prediction error; a concept implemented within the error‐driven learning account of Kruschke (2001b). Therefore, this study both furthers the neuroscientific literature investigating prediction error and strongly supports the account implemented within Kruschke's EXIT formal model.

## AUTHOR CONTRIBUTIONS

**Angus B. Inkster**: Lead author on all aspects including write‐up. **Fraser Milton**: Assistance with fMRI analysis, interpretation, and write‐up. **Charlotte E. R. Edmunds**: Assistance with programming, data collection, and write‐up. **Abdelmalek Benattayallah**: Radiography support. **Andy J. Wills**: Experimental design, plus assistance with behavioral analysis, interpretation, and write‐up.

## Data Availability

The raw imaging and behavioral data, as well as the analysis and modeling scripts for the experiment within this article are available at https://osf.io/yw6fj/.

## Endnotes

^1^
O'Bryan et al. report that the proportion of rare responding to BC (.5) is significantly greater than the base‐rate of .25. This supports the presence of base‐rate neglect, but lacks the greater rare compared to common responding indicative of an IBRE. Similarly, their demonstration of significantly greater rare responding to BC compared to rare responding to A suggests base‐rate neglect is smaller for A than BC, but does not show the presence of an IBRE.

^2^
The slight difference in mean duration relative to the training phase results from discretizing the exponential distribution of times over a different, finite, number of trials.

## Modeling

The simulation was conducted using *slpEXIT*, part of the *catlearn* R package (Wills et al., 2019). This implementation of EXIT is based on the model as described in Kruschke (2001b), with the inclusion of a bias cue that was later implemented in Kruschke (2003). The salience of the bias cue is represented by the σ parameter.

The EXIT model was applied to simulated training and test trials that replicated the details of the experimental procedure, generating response patterns for each simulated trial. The values of the free parameters given to the model were optimized using the *optim* function in R (R Core Team, 2018); specifically the limited memory Broyden–Fletcher–Goldfarb–Shanno algorithm (Byrd, Lu, Nocedal, & Zhu, 1995). The sum of squared errors (SSE) between the model predictions and behavioral data was used as the objective function. As *optim* requires an initial set of starting parameters to vary, each free parameter within the EXIT model was initially set to one of two values. As there are seven free parameters, this resulted in a total of $2^{7}$ or 128 sets of parameter values. This produced 128 sets of optimized parameter values; the set with the lowest SSE was chosen. The parameter values within this final optimized set were: $c=.746,P=2.383,\phi =2.963,\lambda_{g}=.257,\lambda_{w}=.047,\lambda_{x}=2.069,\sigma =.031$.
